# Construction and validation of a prediction model for developing type 2 diabetes mellitus in patients with chronic obstructive pulmonary disease

**DOI:** 10.3389/fendo.2025.1560631

**Published:** 2025-08-15

**Authors:** Xi Kang, Tianye Li, Qinyang Chen, Hao Xu, Yanqiu Jiang, Hongjun Zhao, Xuhong Chang

**Affiliations:** ^1^ School of Public Health, Lan Zhou University, Lanzhou, China; ^2^ Department of Respiratory and Critical Care Medicine, The First Affiliated Hospital of Wenzhou Medical University, Wenzhou, China; ^3^ Zhejiang Province Engineering Research Center for Endoscope Instruments and Technology Development, Clinical Research Center, Department of Pulmonary and Critical Care Medicine, Quzhou People’s Hospital, The Quzhou Affiliated Hospital of Wenzhou Medical University, Quzhou, China

**Keywords:** chronic obstructive pulmonary disease, type 2 diabetes mellitus, complication, prediction, nomogram

## Abstract

**Background:**

Type 2 diabetes mellitus (T2DM) is a common comorbidity of chronic obstructive pulmonary disease (COPD), which significantly increases the risk of rehospitalization and mortality in patients with COPD. Therefore, the purpose of this study was to identify the influencing factors of COPD complicated by T2DM and to construct a visualized disease prediction model.

**Method:**

We included the medical records of 1,773 patients with COPD treated at Quzhou People’s Hospital from 2020 to 2023. Subjects were randomly divided into a training set (n = 1,241) and a test set (n = 532) in a 7:3 ratio. Variable selection was performed using the least absolute shrinkage and selection operator (LASSO), Pearson correlation, and multicollinearity diagnostics. Variables were then refined through backward stepwise selection based on the Akaike Information Criterion (AIC) to construct a nomogram. The accuracy of the nomogram was evaluated using receiver operating characteristic (ROC) curves, calibration curves, and the Hosmer–Lemeshow test (H-L test). The clinical utility of the model was evaluated using decision analysis curves (DCA). Additionally, k-fold cross-validation (k = 10) was performed to rigorously assess model stability and mitigate the risk of overfitting. A sex-stratified subgroup analysis was also conducted to address potential sex-related bias.

**Results:**

The prevalence of T2DM in COPD patients was 27.13%. Seven independent predictors of COPD complicated by T2DM were identified: arterial partial pressure of carbon dioxide (PCO_2_) (OR = 1.04, 95%CI: 1.02–1.05), neutrophil number (NEUT) (OR = 1.15, 95%CI: 1.10–1.19), C-reactive protein (CRP) (OR = 1.01, 95%CI: 1.01–1.02), erythrocyte sedimentation rate (ESR) (OR = 1.03, 95%CI: 1.02–1.05), bilirubin (OR = 0.92, 95%CI: 0.88–0.96), triglyceride (TG) (OR = 1.33, 95%CI: 1.13–1.56), and body mass index (BMI) (OR = 1.16, 95%CI: 1.11–1.20). The model demonstrated good predictive performance, with a C-index of 0.78. The area under the curve (AUC) values were 0.79 (95%CI: 0.76–0.81) for the training set and 0.80 (95%CI: 0.76–0.84) for the test set, consistent with the k-fold cross-validation average AUC of 0.79 (95%CI: 0.76–0.81). Calibration curves and the H-L test (*P >*0.05) indicated good agreement between predicted and observed outcomes. DCA curves demonstrated clinical utility across threshold probabilities. Subgroup analysis showed robust performance in both male (0.82, 95%CI: 0.77–0.86) and female (0.71, 95%CI: 0.60–0.83) groups, with no significant difference in discriminatory ability (DeLong *P* = 0.101).

**Conclusion:**

In this study, we developed and internally validated a visualized prediction model for early identification of T2DM risk in patients with COPD. This tool may facilitate targeted prevention strategies by identifying high-risk populations. While the model demonstrated good performance, external validation is still required to confirm its generalizability.

## 1 Introduction

Chronic obstructive pulmonary disease (COPD) is the third leading cause of death worldwide (1). It affects over 200 million people and causes approximately 3.3 million deaths annually, with both prevalence and mortality rates increasing rapidly (2). Among individuals aged 30–79, the prevalence of COPD had reached 10.3%, and 80.5% of cases occurring in developing countries (3). As the largest developing country in the world, China accounts for 25% of global COPD cases (4), with a prevalence of 8.6% among adults aged ≥20 years and 13.7% among those aged ≥40 years (5). As a major public health challenge, COPD contributes to population disease burden through both widespread prevalence and complex comorbidity profiles. Approximately 40% of COPD patients have one or more complications (6), imposing a substantial disease burden on Chinese society.

COPD increases the risk of developing type 2 diabetes mellitus (T2DM), which is the most common complication, with a prevalence of up to 30% in COPD patients (7). Moreover, complicated by T2DM is associated with a higher risk of 30-day readmission and longer hospital stays in COPD patients (8, 9).This relationship was bidirectional, as T2DM also increases the risk of developing COPD. Evidence suggests that T2DM significantly impairs lung function in COPD patients. For every 1 mmol/L increase in blood glucose levels, there is a reduction of 25 ml in forced vital capacity (FVC) (95%CI: −11 ml to 39 ml), a decrease of 0.71% in FVC percentage (95%CI: −0.34% to 1.08%), a reduction of 13 ml in forced expiratory volume in 1 s (FEV_1_) (95%CI: −2 ml to 25 ml), and a decrease of 0.46% in FEV_1_ percentage (95%CI: −0.09% to 0.83%) (10). Furthermore, the coexistence of COPD and T2DM reduced patients’ exercise capacity and quality of life, increases mortality risk and raises the likelihood of developing other chronic conditions such as heart failure and kidney disease (11–13). Therefore, it was necessary to explore the influencing factors of COPD complicated by T2DM.

Currently, studies increasingly explore the comorbidity mechanisms of COPD and T2DM and observe that chronic inflammation may link them. High levels of inflammatory factors in COPD, such as C-reactive protein (CRP) and tumor necrosis factor α (TNF-α), are considered risk factors for developing T2DM (14). In addition, oxidative stress (15, 16) and obesity (17) are other potential risk factors. Studies had found that excess oxidative substances and adipocytes could promote the production of inflammatory factors, thereby aggravating systemic inflammation and increasing the risk of T2DM in COPD. However, the mechanisms among these influential factors are complex and have not yet reached a clear consensus in many aspects (16). Moreover, studies on risk prediction models specifically for COPD complicated by T2DM are relatively scarce, limiting our ability to fully understand and effectively manage this disease.

Consequently, this study utilized least absolute shrinkage and selection operator (LASSO) regression and mutivariable logistic regression to identify potential factors influencing the occurrence of T2DM in COPD patients. Additionally, we constructed a nomogram to facilitate early diagnosis and intervention. This model has clinical application value by promoting more precise screening, prevention, and management of high-risk populations for COPD complicated by T2DM, thereby enhancing the cost-effectiveness of public health resource utilization.

## 2 Methods

### 2.1 Study population

This retrospective case–control study utilized data from patients treated in the Department of Respiratory and Critical Care Medicine at Quzhou People’s Hospital from 2020 to 2023. The sample size was calculated based on the events per variable (EPV) criterion (18). We anticipated incorporating seven core predictor variables into the model and adopted a conservative EPV threshold of 20. The training set was allocated 70% of the data, while a 10% buffer was reserved for invalid samples to account for data omissions or quality-related exclusions. Consequently, a minimum final sample size of 223 cases was required.

**Figure 1:**
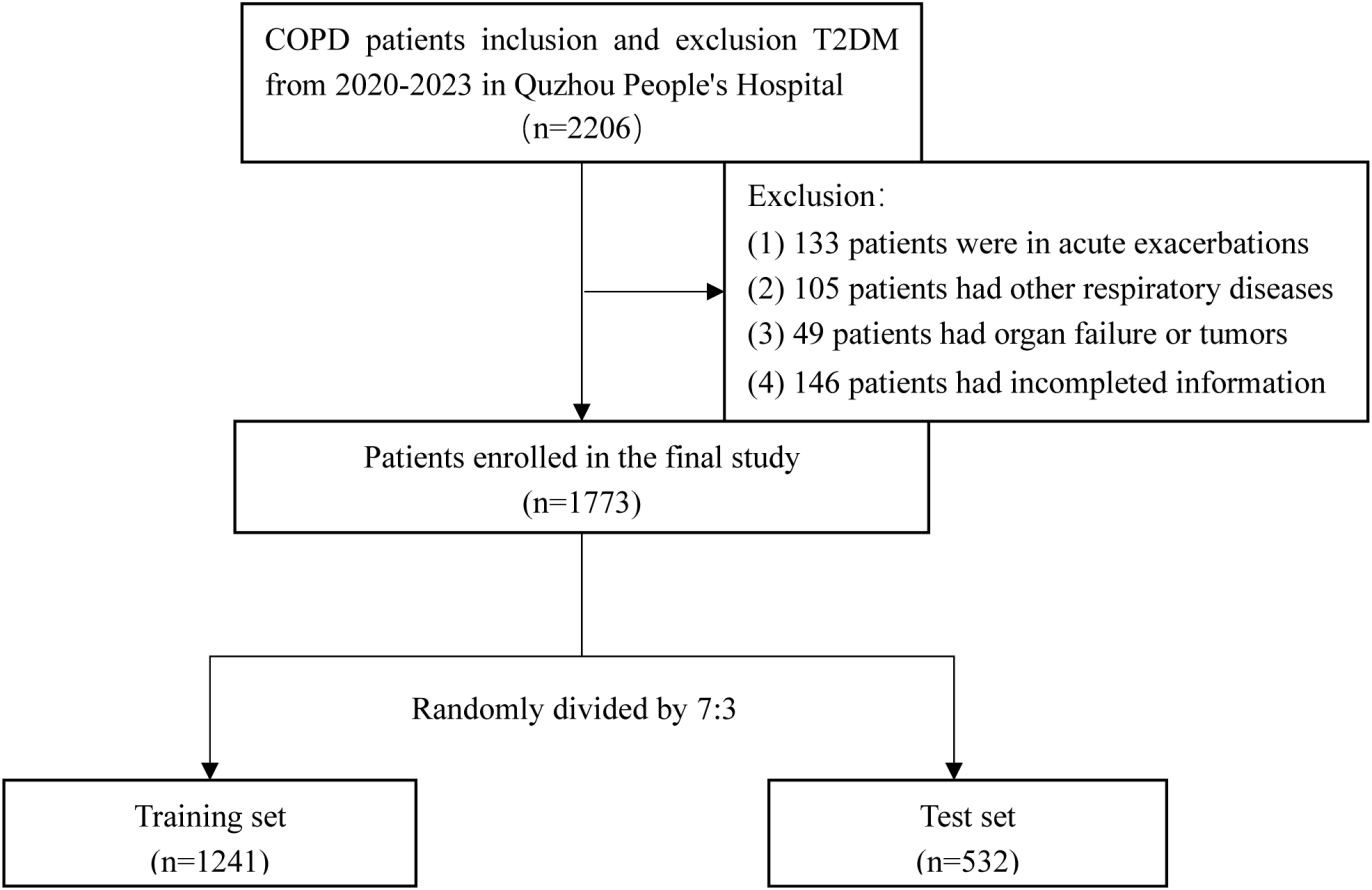
Screening process for subjects in this study.

After screening, 1,773 patients were included, and the specific screening process is shown in **Figure 1**. COPD diagnosis followed the Global Initiative for Chronic Obstructive Lung Disease (GOLD) guidelines (19): a prior clinical COPD diagnosis or post-bronchodilator forced expiratory volume in 1 s (FEV1)/forceful lung volume (FVC) <70% with a stable clinical profile for ≥3 months. T2DM patients diagnosis required (1): a previous clinical diagnosis (2), random blood glucose level ≥11.1 mmol/L (3), fasting blood glucose level ≥7.0 mmol/L, or (4) blood glucose level ≥11.1 mmol/L at 2 h after glucose loading, according to the Guidelines for the Prevention and Control of T2DM (20). The study was approved by the Ethics Committee of Quzhou People’s Hospital (2024–122) and conducted in accordance with the Declaration of Helsinki.

### 2.2 Inclusion and exclusion criteria

Inclusion criteria (1): Age ≥18 years (2); clinically stable (no exacerbations for ≥3 months) (3); complete laboratory and test data (4); provided signed informed consent and demonstrated good compliance. Exclusion criteria: Patients were excluded if they met any of the following conditions (1): Presence of respiratory diseases other than COPD, such as interstitial pneumonia or asthma (2); diagnosis of type 1 diabetes mellitus (3); presence of acute or chronic complications (4); comorbidities including thyroid disease, severe liver or renal insufficiency, malignancies, recent surgery, or major trauma.

### 2.3 Clinical data collection

Patient demographics collected included: age, sex, height, weight, body mass index (BMI), past medical history, history of smoking and alcohol consumption. Admission laboratory tests indicators included: neutrophil count (NEUT, ×10^9^/L), C-reactive protein (CRP, mg/L), erythrocyte sedimentation rate (ESR, mm/h), partial pressure of carbon dioxide (PCO_2_, mmHg), total cholesterol (TC, mmol/L), triglycerides (TG, mmol/L), high-density lipoprotein (HDL, mmol/L), low-density lipoprotein (LDL, mmol/L), total bilirubin (μmol/L), albumin (Alb, g/L), globulin (Glb, g/L), hemoglobin (HGB, g/L), platelet count (PLT, ×10^9^/L), D-dimer (mg/L), prothrombin time (PT, S), activated partial thromboplastin time (APTT, S), alanine aminotransferase (ALT, U/L), and aspartate aminotransferase (AST, U/L).

### 2.4 Statistical analysis

The normality of continuous variables was assessed using the Kolmogorov–Smirnov test. Normally distributed variables were described by mean (SD) and compared between groups using Student’s t-test, while non-normally distributed variables were described by median (Q1, Q3) and compared using the Mann–Whitney U test. Categorical variables were presented as n (%) and compared using the chi-square test or Fisher’s exact test.

The study population was randomly divided into training and test sets in a 7:3 ratio. Mann–Whitney U test, Student’s t-test, and chi-square test were used to compare differences in clinical indicators between the two sets and two groups. In the training set, variables were initially selected using LASSO regression. Subsequently, the variables identified by LASSO regression underwent further screening using Pearson correlation analysis (r <0.7) and multicollinearity diagnosis (VIF <4). The resulting variables were incorporated into a multivariate logistic regression model. The final diagnostic model of COPD combined with T2DM was constructed using backward stepwise selection based on the Akaike Information Criterion (AIC), and a nomogram was developed. The diagnostic model was evaluated using receiver operating characteristic curves (ROC), calibration curves, the Hosmer–Lemeshow test (H-L test), and decision curve analysis (DCA), and was internally validated using the test set. To further evaluate the robustness of the prediction model and address potential overfitting concerns inherent in a single data split, we performed k-fold cross-validation (k = 10) on the entire dataset. In addition, we conducted subgroup analysis to evaluate the model’s performance in male and female subgroups, thereby further validating the generalizability of the predictive model.

Statistical analyses were performed using SPSS version 26.0 and R version 4.4.2, and results were considered statistically significant at a two-sided *P <*0.05.

## 3 Results

### 3.1 Clinical characteristics of the study population

A total of 1,773 COPD patients were included in this study, of whom 481 (27.13%) were also diagnosed with T2DM. The average age of the patients was 72.32 years, with 262 (14.78%) females and 1,511 (85.22%) males. All patients were randomly divided into a training set (n = 1,241) and a test set (n = 532) in a 7:3 ratio. Variable characteristics showed no significant differences between the two sets (*P >*0.05, **Table 1**).

**Table 1:** The equilibrium test of the training set and test set.

Variables	Total (n = 1,773)	Test (n = 532)	Training (n = 1,241)	Statistic	*P*
Age, Mean ± SD	72.32 ± 9.22	72.55 ± 9.02	72.22 ± 9.31	0.68^*^	0.497
Height, Mean ± SD	164.46 ± 7.67	164.28 ± 7.80	164.53 ± 7.61	−0.62^*^	0.534
Weight, Mean ± SD	58.96 ± 11.39	58.97 ± 11.38	58.96 ± 11.39	0.02^*^	0.981
BMI, Mean ± SD	21.79 ± 3.86	21.86 ± 3.96	21.76 ± 3.82	0.53^*^	0.599
With T2DM, n(%)				0.01^**^	0.924
No	1,292 (72.87)	393 (73.87)	899 (72.44)		
Yes	481 (27.13)	139 (26.13)	342 (27.56)		
Smoke, n(%)				0.27^**^	0.605
No	687 (38.75)	211 (39.66)	476 (38.36)		
Yes	1,086 (61.25)	321 (60.34)	765 (61.64)		
Drink, n(%)				3.38^**^	0.066
No	1,133 (63.90)	357 (67.11)	776 (62.53)		
Yes	640 (36.10)	175 (32.89)	465 (37.47)		
Gender, n(%)				0.41^**^	0.522
Female	262 (14.78)	83 (15.60)	179 (14.42)		
Male	1,511 (85.22)	449 (84.40)	1,062 (85.58)		
Past medical history, n(%)				0.70^**^	0.403
No	1,457 (82.18)	431 (81.02)	1,026 (82.68)		
Yes	316 (17.82)	101 (18.98)	215 (17.32)		
PLT, M (Q_1_, Q_3_)	223.00 (174.00, 275.00)	227.50 (178.75, 280.00)	221.00 (173.00, 274.00)	−1.80^***^	0.072
HGB, M (Q_1_, Q_3_)	30.40 (29.10, 32.80)	30.40 (29.10, 33.52)	30.30 (29.00, 32.50)	−0.70^***^	0.484
PCO_2_, M (Q_1_, Q_3_)	47.70 (44.90, 52.30)	47.70 (45.25, 52.30)	47.70 (44.80, 52.30)	−0.29^***^	0.772
NEUT, M (Q_1_, Q_3_)	5.27 (3.73, 7.82)	5.24 (3.63, 7.84)	5.28 (3.77, 7.79)	-0.73^***^	0.466
ESR, M (Q_1_, Q_3_)	24.00 (22.00, 33.00)	24.00 (21.00, 33.00)	24.00 (22.00, 33.00)	−0.78^***^	0.438
CRP, M (Q_1_, Q_3_)	18.70 (14.40, 31.40)	18.70 (13.53, 31.40)	18.70 (14.60, 31.40)	−1.18^***^	0.239
D-Dimer, M (Q_1_, Q_3_)	0.78 (0.38, 1.44)	0.74 (0.37, 1.50)	0.79 (0.38, 1.42)	−0.15^***^	0.884
PT, M (Q_1_, Q_3_)	13.50 (13.00, 14.20)	13.50 (12.90, 14.20)	13.50 (13.00, 14.20)	−0.59^***^	0.555
APTT, M (Q_1_, Q_3_)	38.10 (34.90, 42.00)	38.50 (35.10, 42.60)	38.10 (34.90, 41.70)	−1.14^***^	0.256
ALT, M (Q_1_, Q_3_)	18.00 (12.00, 26.00)	18.00 (12.00, 26.00)	18.00 (12.00, 26.00)	−0.61^***^	0.541
AST, M (Q_1_, Q_3_)	22.00 (18.00, 28.00)	22.00 (18.00, 27.00)	22.00 (18.00, 28.00)	−0.12^***^	0.905
Alb, M (Q_1_, Q_3_)	36.10 (32.80, 39.10)	36.40 (33.20, 39.00)	36.00 (32.60, 39.20)	−1.05^***^	0.293
Glb, M (Q_1_, Q_3_)	29.90 (26.80, 33.60)	30.00 (26.90, 33.73)	29.90 (26.70, 33.60)	−0.80^***^	0.422
Bilirubin, M (Q_1_, Q_3_)	10.00 (8.00, 12.00)	10.00 (8.00, 12.00)	10.00 (8.00, 12.00)	−1.14^***^	0.256
TC, M (Q_1_, Q_3_)	4.43 (3.61, 5.46)	4.47 (3.64, 5.41)	4.43 (3.58, 5.47)	−0.41^***^	0.685
TG, M (Q_1_, Q_3_)	1.16 (0.89, 1.63)	1.17 (0.89, 1.65)	1.15 (0.89, 1.62)	−0.26^***^	0.795
HDL, M (Q_1_, Q_3_)	1.06 (0.93, 1.23)	1.08 (0.95, 1.23)	1.05 (0.92, 1.23)	−1.11^***^	0.269
LDL, M (Q_1_, Q_3_)	2.84 (2.33, 3.30)	2.84 (2.37, 3.30)	2.84 (2.31, 3.30)	−0.19^***^	0.849

*t-test, **Chi-square test, ***Mann–Whitney test.

SD, standard deviation; M, Median; Q_1_, 1st Quartile; Q_3_, 3rd Quartile.

In both the training and test sets, the COPD with T2DM group exhibited significantly higher levels of inflammatory markers compared to the COPD without T2DM group, including NEUT (training set: 6.83 vs 4.82, *P <*0.001; test set: 6.65 vs 4.83, *P <*0.001), ESR (training set: 33.00 vs 24.00, *P <*0.001; test set: 33.00 vs 24.00, *P <*0.001), and CRP (training set: 31.40 vs 18.50, *P <*0.001; test set: 31.40 vs 18.50, *P <*0.001). Blood lipid profiles also differed significantly, with elevated TG (training set: 1.46 vs 1.08, *P <*0.001; test set: 1.51 vs 1.10, *P <*0.001) and LDL (training set: 3.00 vs 2.81, *P <*0.001; test set: 3.16 vs 2.80, *P <*0.001), but reduced HDL (training set: 0.99 vs 1.14, *P <*0.001; test set: 1.00 vs 1.16, *P <*0.001). Additionally, the COPD with T2DM group showed higher blood gas indices, specifically PCO_2_ (training set: 51.80 vs 47.70, *P <*0.001; test set: 51.90 vs 47.70, *P <*0.001). Conversely, total bilirubin levels (training set: 9.00 vs 11.00, *P <*0.001; test set: 9.00 vs 11.00, *P <*0.001) were significantly lower in the COPD with T2DM group (**Table 2**).

**Table 2:** Analysis of clinical features in COPD with T2DM and only COPD patients.

Factors	Training				Test			
	Without T2DM (n = 899)	With T2DM (n = 342)	Statistic	*P*	Without T2DM (n = 393)	With T2DM (n = 139)	Statistic	*P*
Age, Mean ± SD	71.42 ± 9.26	74.34 ± 9.11	−4.98^*^	<.001	71.75 ± 9.17	74.82 ± 8.19	−3.49^*^	<.001
Height, Mean ± SD	164.24 ± 7.57	165.30 ± 7.68	−2.19^*^	0.029	164.25 ± 7.85	164.37 ± 7.66	−0.15^*^	0.881
Weight, Mean ± SD	57.59 ± 10.47	63.54 ± 11.66	−8.26^*^	<.001	57.79 ± 11.34	63.46 ± 12.42	−4.94^*^	<.001
BMI, Mean ± SD	21.35 ± 3.72	23.21 ± 3.69	−7.85	<.001	21.39 ± 3.73	23.49 ± 4.36	−5.45^*^	<.001
Smoke, n (%)			2.39^**^	0.122			17.73^**^	<.001
No	333 (37.04)	143 (41.81)			135 (34.35)	76 (54.68)		
Yes	566 (62.96)	199 (58.19)			258 (65.65)	63 (45.32)		
Drink, n (%)			0.01^**^	0.911			3.36^**^	0.067
No	563 (62.63)	213 (62.28)			255 (64.89)	102 (73.38)		
Yes	336 (37.37)	129 (37.72)			138 (35.11)	37 (26.62)		
Gender, n (%)			10.21^**^	0.001			19.68^**^	<.001
Female	112 (12.46)	67 (19.59)			45 (11.45)	38 (27.34)		
Male	787 (87.54)	275 (80.41)			348 (88.55)	101 (72.66)		
Past medical history, n (%)			24.11^**^	<.001			13.11^**^	<.001
No	714 (79.42)	312 (91.23)			304 (77.35)	127 (91.37)		
Yes	185 (20.58)	30 (8.77)			89 (22.65)	12 (8.63)		
PLT, M (Q_1_, Q_3_)	222.00 (174.50, 273.00)	221.00 (163.25, 274.75)	−1.08^***^	0.279	230.00 (185.00, 283.00)	222.00 (166.50, 271.50)	−1.33^***^	0.184
HGB, M (Q_1_, Q_3_)	30.40 (29.10, 32.20)	30.30 (28.90, 103.00)	−0.72^**^	0.473	30.40 (29.30, 33.00)	30.00 (28.50, 68.35)	−2.28^***^	0.023
PCO_2_, M (Q_1_, Q_3_)	47.70 (43.70, 51.10)	51.80 (49.60, 52.80)	−9.06^**^	<.001	47.70 (44.40, 51.70)	51.90 (49.60, 53.10)	−6.00^***^	<.001
NEUT, M (Q_1_, Q_3_)	4.82 (3.50, 7.17)	6.83 (4.85, 9.05)	−9.44^***^	<.001	4.83 (3.35, 6.93)	6.65 (5.04, 9.34)	−6.35^***^	<.001
ESR, M (Q_1_, Q_3_)	24.00 (20.00, 33.00)	33.00 (32.50, 33.00)	−11.06^***^	<.001	24.00 (17.00, 32.50)	33.00 (32.50, 33.00)	−7.40^***^	<.001
CRP, M (Q_1_, Q_3_)	18.50 (12.80, 26.72)	31.40 (25.12, 31.60)	−11.55^***^	<.001	18.50 (12.66, 23.20)	31.40 (22.38, 31.65)	−7.51^***^	<.001
D-Dimer, M (Q_1_, Q_3_)	0.75 (0.37, 1.30)	0.98 (0.45, 1.60)	−3.72^***^	<.001	0.63 (0.35, 1.30)	1.01 (0.51, 1.81)	−3.42^***^	<.001
PT, M (Q_1_, Q_3_)	13.50 (13.00, 14.20)	13.50 (13.00, 14.30)	−0.95^***^	0.342	13.50 (13.00, 14.10)	13.50 (12.90, 14.30)	−0.02^***^	0.984
APTT, M (Q_1_, Q_3_)	38.30 (35.00, 42.15)	37.70 (34.62, 41.08)	−2.15^***^	0.031	38.80 (35.30, 42.60)	37.60 (34.50, 42.75)	−1.19^***^	0.234
ALT, M (Q_1_, Q_3_)	17.00 (12.00, 26.00)	20.00 (14.00, 25.00)	−2.57^***^	0.010	17.00 (12.00, 24.00)	22.00 (14.50, 30.00)	−4.04^***^	<.001
AST, M (Q_1_, Q_3_)	22.00 (18.00, 28.00)	22.00 (18.00, 27.00)	−0.03^***^	0.974	21.00 (18.00, 27.00)	24.00 (19.00, 28.00)	−2.13^***^	0.033
Alb, M (Q_1_, Q_3_)	36.20 (32.95, 39.50)	35.00 (31.80, 39.00)	−2.58^***^	0.010	36.80 (33.60, 39.20)	35.20 (32.45, 38.50)	−2.65^***^	0.008
Glb, M (Q_1_, Q_3_)	29.90 (26.80, 33.60)	29.75 (26.60, 33.45)	−0.26^***^	0.791	29.80 (26.70, 33.70)	30.30 (27.00, 33.85)	−0.61^***^	0.544
Bilirubin, M (Q_1_, Q_3_)	11.00 (8.00, 12.00)	9.00 (8.00, 10.00)	−4.94^***^	<.001	11.00 (8.00, 12.00)	9.00 (8.00, 10.00)	−3.75^***^	<.001
TC, M (Q_1_, Q_3_)	4.35 (3.56, 5.32)	4.66 (3.74, 5.51)	−1.69^***^	0.092	4.31 (3.61, 5.25)	4.86 (3.73, 5.52)	−2.01^***^	0.044
TG, M (Q_1_, Q_3_)	1.08 (0.86, 1.54)	1.46 (1.02, 1.77)	−6.49^***^	<.001	1.10 (0.86, 1.53)	1.51 (1.04, 1.77)	−4.10^***^	<.001
HDL, M (Q_1_, Q_3_)	1.14 (0.92, 1.28)	0.99 (0.91, 1.10)	−6.87^***^	<.001	1.16 (0.95, 1.26)	1.00 (0.94, 1.08)	−5.16^***^	<.001
LDL, M (Q_1_, Q_3_)	2.81 (2.26, 3.30)	3.00 (2.44, 3.33)	−3.73^***^	<.001	2.80 (2.33, 3.19)	3.16 (2.53, 3.49)	−4.37^***^	<.001

*t-test, **Chi-square test, ***Mann–Whitney test.

SD, standard deviation; M, Median; Q_1_, 1st Quartile; Q_3_, 3rd Quartile.

### 3.2 Selection of study variables

**Figure 2:**
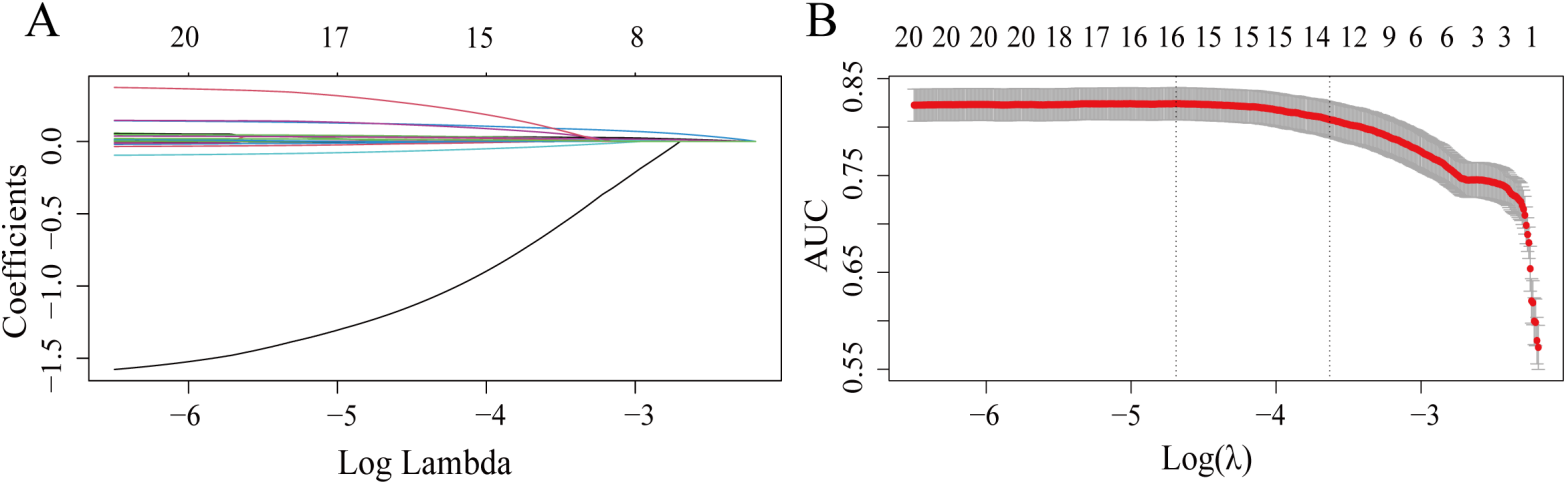
Lasso regression variable selection. **(A)** Number of retained features across log (λ) values; **(B)** Coefficient shrinkage paths with AUC (top axis).

The 26 variables in the training set underwent variable selection via LASSO regression (**Figure 2A**), and coefficient changes for each variable were shown in **Figure 2B**. Using 10-fold cross-validation, the optimal model was obtained at λ = 0.021 (Log(λ) = −3.86). A total of 14 variables were selected: PLT, HGB, APTT, NEUT, CRP, ESR, PCO_2_, HDL, LDL, TG, bilirubin, weight, BMI, and age.

Subsequent Pearson correlation analysis and multicollinearity diagnostics revealed strong collinearity between BMI and weight (r = 0.865,VIF >4). Given BMI’s superior utility in quantifying obesity, weight was excluded (**Table 3**).

**Table 3:** Pearson correlation and multicollinearity analysis among variables.

Variables	PLT	HGB	APTT	NEUT	PCO2	Bilirubin	HDL	CRP	TG	Weight	ESR	Age	LDL	BMI	VIF
PLT	1														1.167
HGB	−0.163^**^	1													1.061
APTT	0.084^**^	0.007	1												1.060
NEUT	0.151^**^	0.095^**^	0.017	1											1.083
PCO_2_	−0.087^**^	0.031	−0.050	0.105^**^	1										1.037
Bilirubin	−0.087^**^	0.051	0.069^*^	−0.020	−0.024	1									1.043
HDL	−0.058^*^	−0.017	−0.049	−0.062^**^	−0.020	−0.092^**^	1								1.103
CRP	0.116^**^	−0.032	0.155^**^	0.141^**^	0.060^*^	0.005	−0.070^*^	1							1.172
TG	−0.032	−0.080^**^	−0.082^**^	0.011	−0.027	−0.075^**^	−0.087^**^	−0.091^**^	1						1.112
Weight	−0.091^**^	0.051	0.039	0.056^*^	−0.045	−0.034	−0.120^**^	−0.047	0.118^**^	1					4.066
ESR	0.247^**^	−0.056^*^	0.152^**^	0.139^**^	0.008	−0.071^*^	−0.129^**^	0.331^**^	−0.019	0.024	1				1.227
Age	−0.096^**^	0.054	0.034	−0.009	0.011	0.043	−0.032	0.005	−0.057^*^	−0.044	−0.006	1			1.025
LDL	0.068^*^	−0.051	−0.053	0.004	−0.061^*^	−0.092^**^	0.166^**^	−0.070^*^	0.239^**^	0.049	0.056	−0.036	1		1.132
BMI	−0.106^**^	0.073^*^	0.026	0.038	−0.028	−0.019	−0.097^**^	−0.066^*^	0.131^**^	0.865^**^	−0.008	−0.011	0.056	1	4.043

*p <0.05,** p <0.01.

### 3.3 Construction of the nomogram

Subsequently, 13 candidate variables were retained for logistic regression analysis. Backward stepwise selection based on AIC further refined the model, resulting in seven independent predictors. The final model demonstrated good discrimination (C-index = 0.78; **Table 4**).

**Table 4:** Multivariate logistic analysis for influence factors in patients with COPD and T2DM.

Variables	β	S.E	Z	*P*	OR (95%CI)
Intercept	−7.75	0.70	−10.56	<.001	–
PCO_2_	0.04	0.01	4.91	<.001	1.04 (1.02–1.05)
NEUT	0.14	0.02	6.65	<.001	1.15 (1.10–1.19)
CRP	0.01	0.004	3.40	<.001	1.01 (1.01–1.02)
ESR	0.03	0.01	6.41	<.001	1.03 (1.02–1.05)
Bilirubin	−0.09	0.02	−3.99	0.046	0.92 (0.88–0.96)
TG	0.29	0.08	3.43	<.001	1.33 (1.13–1.57)
BMI	0.05	0.02	7.56	<.001	1.16 (1.11–1.20)

OR, Odds Ratio; CI, Confidence Interval.

The model indicated that elevated levels of PCO_2_ (OR = 1.04, 95%CI: 1.02–1.05), NEUT (OR = 1.15, 95%CI: 1.10–1.19), CRP (OR = 1.01, 95%CI: 1.01–1.02), ESR (OR = 1.03, 95%CI: 1.02–1.05), TG (OR = 1.33, 95%CI: 1.13–1.56), and BMI (OR = 1.16, 95%CI: 1.11–1.20) were significantly associated with an increased risk of COPD combined with T2DM. Conversely, a higher level of bilirubin (OR = 0.92, 95%CI: 0.88–0.96) was associated with a decreased risk of developing COPD complicated with T2DM.

**Figure 3:**
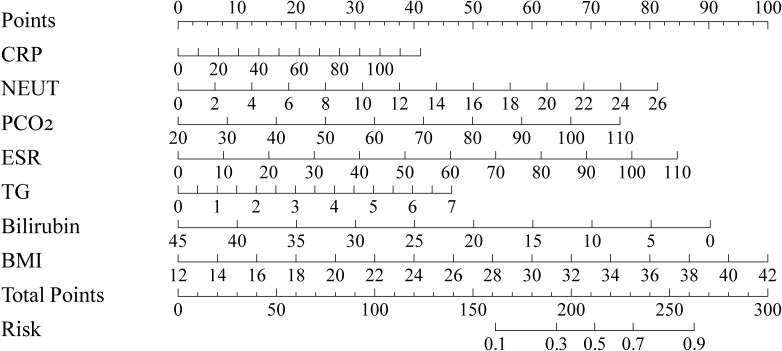
Nomogram for COPD with T2DM reserving CRP, NEUT, PCO2, ESR, TG, Bilirubin,BMI as predictors.

**Figure 4:**
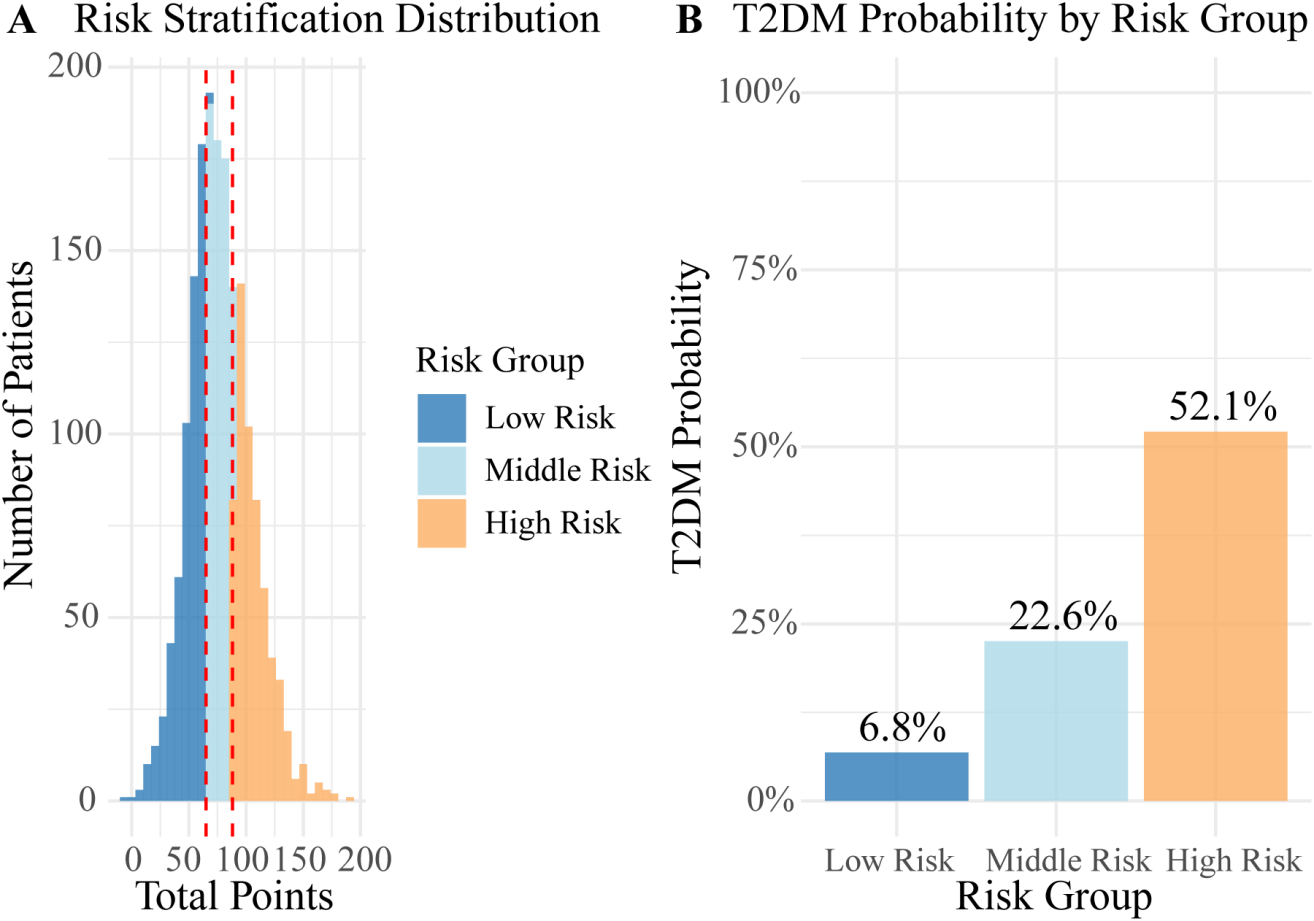
Three-level risk stratification of the predictive model. **(A)** Patient distribution across risk groups; **(B)** T2DM probability per risk group (Low: 6.8%, Middle: 22.6%, High: 52.1%).

A nomogram was constructed by assigning scores proportional to the regression coefficients (**Figure 3**). Summing the individual scores yields a total point value, which corresponds to the T2DM risk probability on the bottom axis. Based on total scores, a risk classification system was established, categorizing patients into three groups (**Figure 4**): low-risk (score: 0.0–64.9; T2DM probability: 6.8%), medium-risk (score: 65.0–88.1; T2DM probability: 22.6%), and high-risk (score: 88.1–189.2; T2DM probability: 52.1%).

### 3.4 Validation of the nomogram

**Figure 5:**
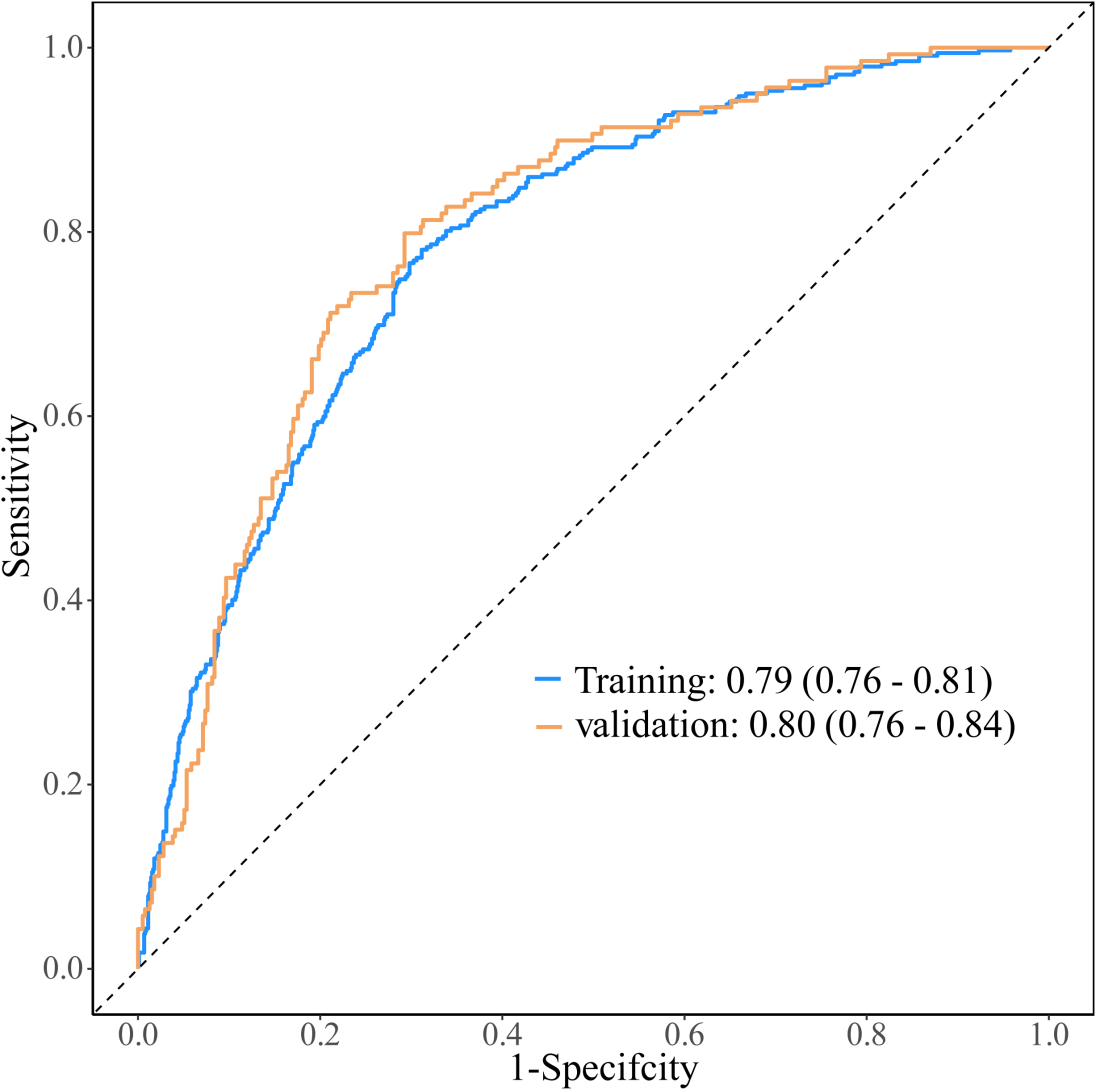
The ROC of the training and test set.

**Figure 6:**
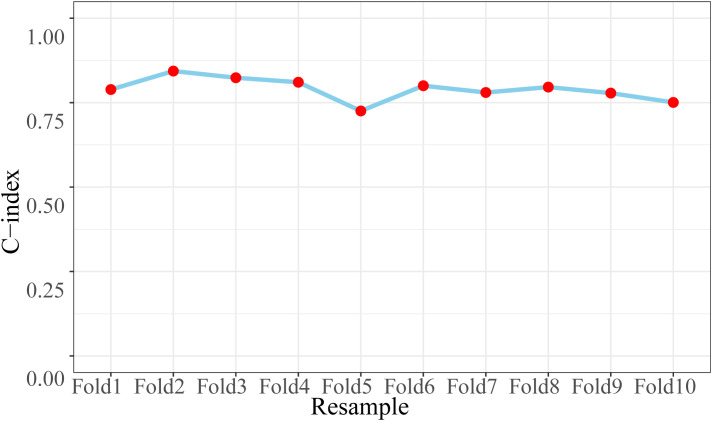
The AUC values across different folds.

The area under the curve (AUC) for the training set and test set was 0.79 (95%CI: 0.76–0.81) and 0.80 (95%CI: 0.76–0.84), respectively (**Figure 5**). To provide a more robust assessment of model performance, k-fold cross-validation was conducted on the entire cohort. The average AUC across the 10 validation folds (**Figure 6**) was 0.79 (95%CI: 0.76–0.81). The consistency of AUC values across folds, along with the average AUC closely aligning with those observed in the independent test set (0.80) and the training set (0.79), demonstrated that the nomogram possessed robust predictive performance and stability.

**Figure 7:**
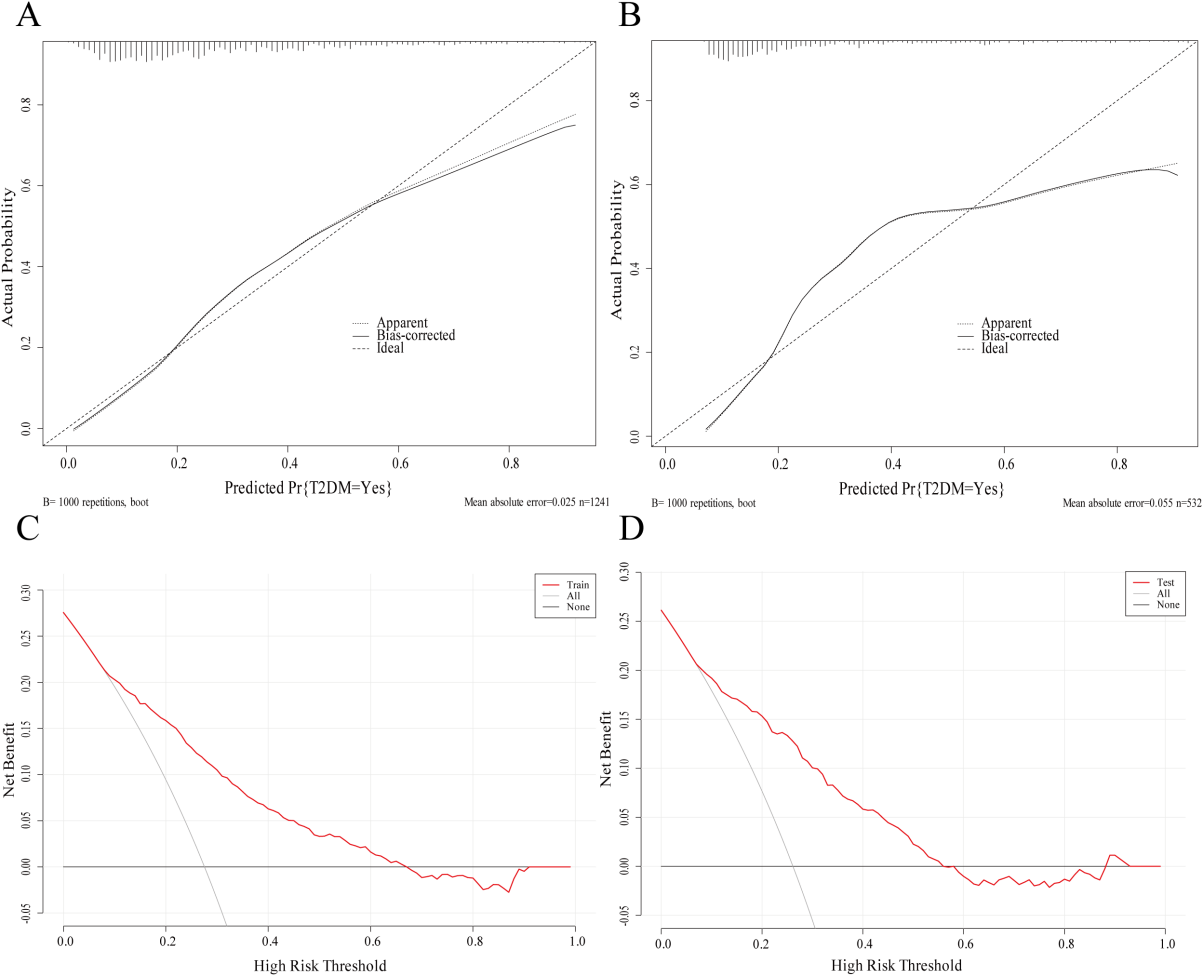
Model validation. **(A)** Calibration curve (training set, n=1241); **(B)** Calibration curve (test set, n=532); **(C)** Decision curve analysis - training set; **(D)** Decision curve analysis - test set.

Calibration curves for the training and test sets indicated good agreement between predicted probabilities and actual outcomes (**Figures 7A, B**). The H-L test showed good calibration (training set: χ² = 10.58, *P* = 0.23; test set: χ² = 12.06, *P* = 0.15). Furthermore, decision curve analysis demonstrated significant clinical utility of the nomogram (**Figures 7C, D**), yielding net clinical benefit across a threshold probability range of 2% to 64% in clinical practice.

**Figure 8:**
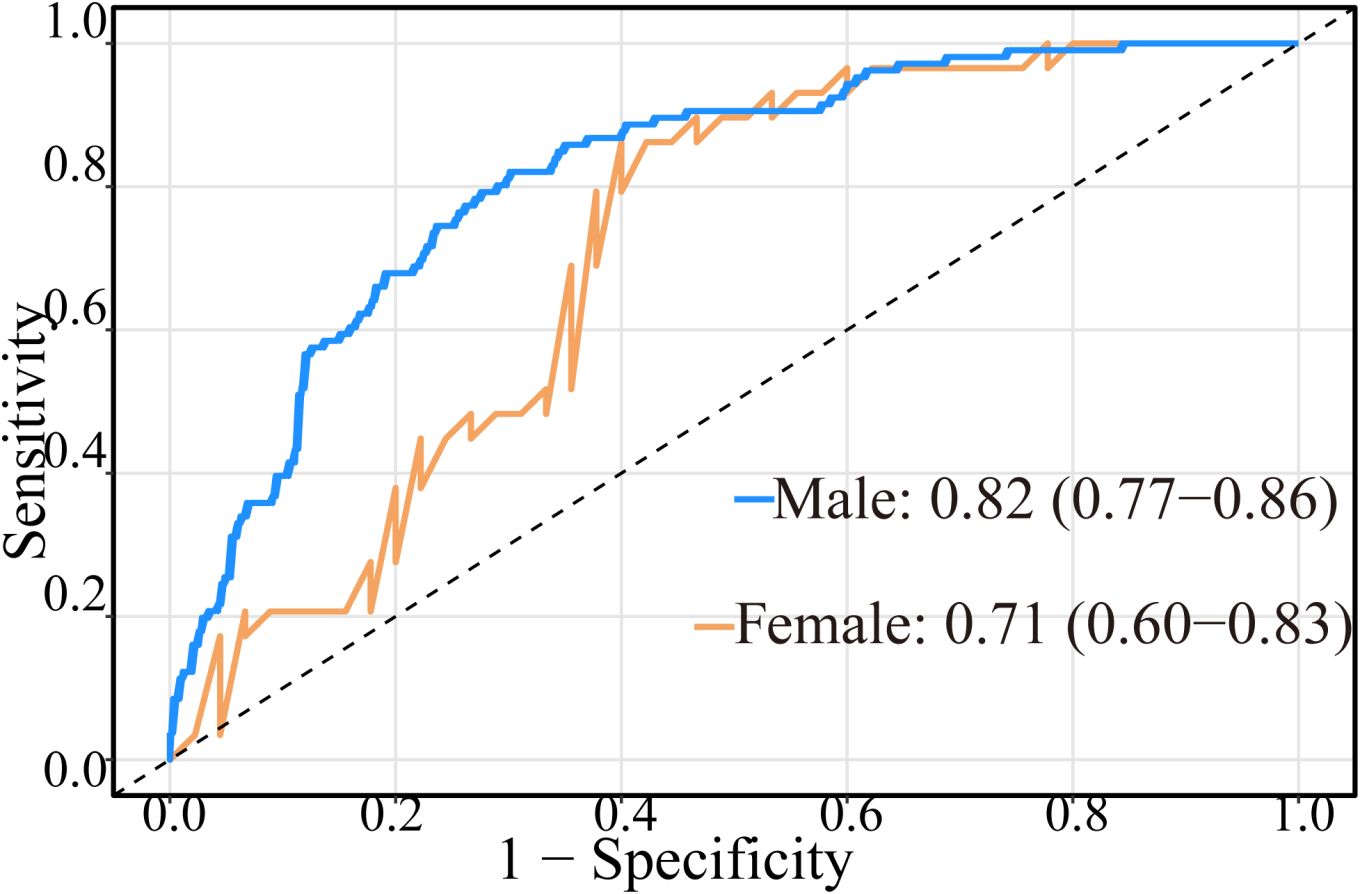
The model validation between male and female subgroups.

Given the imbalanced sex ratio (85.22% male) in our study, we assessed potential sex-related bias to enhance the model’s generalizability (**Figure 8**). Subgroup analysis demonstrated that the model maintained good predictive performance in both sexes (male: 0.82, 95%CI: 0.77–0.86; female: 0.71, 95%CI: 0.60–0.83), with no significant difference in predictive capability between male and female subgroups according to DeLong’s test (*P* = 0.101).

## 4 Discussion

COPD and T2DM are chronic non-communicable diseases of global concern. Substantial evidence indicates that COPD significantly increases the risk of developing T2DM (21, 22). Concurrently, COPD complicated by T2DM also increases the risk of hospital readmission and mortality in patients (8, 9, 23). In this study, we investigated clinical characteristics associated with the prevalence of COPD combined with T2DM and developed an early diagnostic model incorporating PCO_2_, NEUT, CRP, ESR, TG, BMI, and bilirubin. The model demonstrated robust performance and clinical predictive value, offering a novel and reliable tool for early identification of T2DM risk in COPD patients.

Chronic inflammation is a key shared pathophysiological mechanism in COPD and T2DM. Neutrophils, the predominant inflammatory cells in COPD (24), are also elevated in diabetes and served as significant predictors (25, 26). In addition, CRP has been shown to be an independent risk factor for COPD combined with T2DM and to exacerbate insulin resistance (27). Our findings showed that inflammation-related factors were all elevated in the COPD with T2DM group compared to the COPD-only group, with NEUT, CRP, and ESR identified as independent predictors. Elevated peripheral blood neutrophil count is directly proportional to FEV_1_ and emphysema severity (28, 29). Furthermore, neutrophils cause lung tissue damage and worsen lung inflammation by releasing proteases and oxidants. Crucially, enhanced elastase secretion by neutrophils exacerbates diabetic insulin resistance (30). CRP and ESR are also important inflammatory markers in COPD that promote the expression of inflammatory cells during acute exacerbations of COPD symptoms and enhance insulin resistance leading to T2DM. Therefore, early and appropriate anti-inflammatory treatment in COPD patients is crucial.

Our research also revealed that patients with both COPD and T2DM exhibited higher arterial PCO_2_ levels compared to those with COPD alone. Furthermore, T2DM risk increased with rising PCO_2_ levels. This association may be linked to the increased accumulation of advanced glycation end products (AGEs) in the vascular endothelial cells of patients with COPD (31). AGEs stimulate the expression of inflammatory genes by binding to the receptor for advanced glycosylation end products (RAGE) and promote the release of CRP and TNF (32–34). Additionally, AGE–RAGE interactions are more pronounced in the lungs and peripheral airways of COPD patients than in those without COPD (35), leading to thickened lung capillary basement membranes and reduced alveolar ventilation, therey impairing CO_2_ excretion. Consequently, CO_2_ accumulates in the body, elevating arterial PCO_2_ levels. Excessive levels of CO_2_ in the body further stimulate the expression of inflammatory factors through the NF-κB signaling pathway (36), thereby contributing to insulin resistance and increasing T2DM risk. This highlights the importance of preventing CO_2_ retention through early ventilatory support in COPD patients to mitigate T2DM comorbidity.

Obesity, a critical T2DM risk factor, is associated with dyslipidemia. Our study revealed that patients with COPD and T2DM exhibited higher levels of TG and LDL, and lower levels of HDL compared to those with COPD alone. Additionally, we identified increased TG and BMI levels as independent predictive factors for T2DM, corroborating previous findings (37). Reduced physical activity, common in COPD patients due to respiratory limitations and its higher prevalence in older adults (27, 38, 39), promotes glucose intolerance and sarcopenic obesity. Furthermore, substantial evidence suggests that lipids can activate monocytes in the vasculature, enhancing the inflammatory response through the NF-κB and JNK pathways (40, 41) to promote insulin resistance. Consequently, these insights emphasize the need for clinicians to encourage physical activity, a balanced diet, and lipid control in COPD patients early on to reduce T2DM risk.

In addition to inflammation and obesity, oxidative stress significantly contributes to the development of T2DM in COPD. Consistent with prior research (42), our study indicates that serum bilirubin exerts a protective effect against the development of T2DM. Patients with COPD exhibit increased oxidative stress due to exposure to exogenous particles, smoking, and endogenous inflammation. Excessive oxidative stress disrupts the activity of antioxidants such as glutathione (GSH) and decreases the expression of the antioxidant transcription factors erythroid 2-related factor 2 (Nrf2) and the related gene heme oxygenase 1 (HO-1) (43). These molecules are essential for protecting pancreatic β cells from oxidative stress damage (44). At the same time, oxidative stress enhances lung inflammation, creating a vicious cycle. Serum bilirubin is a potent antioxidant that has been shown to be effective in alleviating damage caused by oxidative stress (45). These results suggested that mildly elevated serum bilirubin levels may play an important role in preventing T2DM in COPD patients.

In this study, we identified seven predictors of COPD combined with T2DM, including: PCO2, NEUT, CRP, ESR, bilirubin, TG, and BMI. Our novel predictive model demonstrated strong discriminatory ability and important clinical value. Notably, despite a significant gender imbalance in the study, gender subgroup analysis revealed that the model maintained good predictive performance in both male and female subgroups, with no statistically significant difference in predictive ability between the two groups. This result indicates that the predictive model we established has relatively consistent applicability across patients of different genders, enhancing its potential for clinical translation. From a public health perspective, our findings highlight the substantial increased risk of T2DM in patients with COPD. Our model established a three-level risk stratification based on the total score, with COPD patients in the high-risk group exhibiting a significantly elevated probability of developing T2DM. Consequently, targeted early interventions for the high-risk group are warranted to prevent subsequent hospitalization burdens. Furthermore, this model demonstrates particular suitability for routine screening in COPD outpatient clinics. By inputting patients’ basic blood gas parameters, complete blood count, lipid profile, and BMI data during consultations, clinicians can rapidly obtain individualized risk scores, thereby enhancing clinical resource allocation efficiency.

However, this study had several limitations. First, this study was a single-center study with a limited sample size and no external validation, which might lead to an incomplete understanding of the findings. Although we used a 7:3 random split combined with k-fold cross-validation to minimize overfitting, and our internal validation showed robust performance, we acknowledged that external validation is still the gold standard for assessing model generalizability. Therefore, future research should focus on multi-center studies with external validation to confirm its reproducibility and clinical applicability. Secondly, although our subgroup analysis indicated no statistically significant difference in the model’s predictive capability between male and female patients, the pronounced male predominance in our study raises potential concerns about sex-related differences impacting the generalizability of our model. Current epidemiological evidence suggests that COPD incidence rates are converging between sexes in developed countries (46), potentially linked to changing smoking patterns and differential susceptibility to environmental exposures. Importantly, substantial evidence highlights sex-based dimorphism in inflammatory responses (47). These differences might influence key predictors in our model—particularly neutrophil activation and oxidative stress pathways. While our model demonstrated preliminary utility for risk stratification in both sexes, the limited female sample size precludes definitive exclusion of gender as an effect modifier. Future studies should proactively incorporate gender as a covariate in multivariate modeling and conduct formal interaction analyses to evaluate its role as a potential effect modifier. Furthermore, this was a retrospective study, and the collection of clinical data was incomplete, including pulmonary function indicators, readmission rate, survival and death indicators, which limited the comprehensiveness of the analysis. Therefore, future studies should include prospective cohort studies to explore the accuracy of our findings from multiple perspectives and gain a more complete understanding of the relationship between COPD and T2DM.

## 5 Conclusion

The prediction model based on NEUT, CRP, ESR, PCO_2_, TG, BMI, and bilirubin demonstrated good predictive accuracy and clinical application value in this study. However, its generalizability to broader populations requires external validation and could provide a more scientific reference for the screening and prevention of early COPD complicated with T2DM.

## Data Availability

The raw data supporting the conclusions of this article will be made available by the authors, without undue reservation.
